# Endovascular Versus Medical Therapy in Posterior Cerebral Artery Stroke: Role of Baseline NIHSS Score and Occlusion Site

**DOI:** 10.1161/STROKEAHA.124.047383

**Published:** 2024-05-16

**Authors:** Davide Strambo, Patrik Michel, Thanh N. Nguyen, Mohamad Abdalkader, Muhammad M. Qureshi, Daniel Strbian, Christian Herweh, Markus A. Möhlenbruch, Silja Räty, Marta Olivé-Gadea, Marc Ribo, Marios Psychogios, Urs Fischer, Anh Nguyen, Joji B. Kuramatsu, David Haupenthal, Martin Köhrmann, Cornelius Deuschl, Jordi Kühne Escolà, Jelle Demeestere, Robin Lemmens, Lieselotte Vandewalle, Shadi Yaghi, Liqi Shu, Volker Puetz, Daniel P.O. Kaiser, Johannes Kaesmacher, Adnan Mujanovic, Dominique Cornelius Marterstock, Tobias Engelhorn, Manuel Requena, Hormuzdiyar H. Dasenbrock, Piers Klein, Diogo C. Haussen, Mahmoud H. Mohammaden, Hend Abdelhamid, Lorena Souza Viana, Bruno Cunha, Isabel Fragata, Michele Romoli, Francesco Diana, Wei Hu, Chao Zhang, Pekka Virtanen, Riikka Lauha, Jessica Jesser, Judith Clark, Stavros Matsoukas, Johanna T. Fifi, Sunil A. Sheth, Sergio Salazar-Marioni, João Pedro Marto, João Nuno Ramos, Milena Miszczuk, Christoph Riegler, Sven Poli, Khouloud Poli, Ashutosh P. Jadhav, Shashvat M. Desai, Volker Maus, Maximilian Kaeder, Adnan H. Siddiqui, Andre Monteiro, Hesham E. Masoud, Neil Suryadareva, Maxim Mokin, Shail Thanki, Kemal Alpay, Pauli Ylikotila, James E. Siegler, Italo Linfante, Guilherme Dabus, Negar Asdaghi, Vasu Saini, Christian H. Nolte, Eberhard Siebert, Bettina L. Serrallach, Charlotte S. Weyland, Uta Hanning, Lukas Meyer, Anne Berberich, Peter A. Ringleb, Raul G. Nogueira, Simon Nagel

**Affiliations:** Service of Neurology, Department of Clinical Neurosciences, University Hospital of Lausanne and University of Lausanne, Switzerland (D. Strambo, P.M.).; Neurology (T.N.N., J.C.), Boston Medical Center, MA.; Radiology (T.N.N., M.A., M.M.Q., P.K., H.D.), Boston Medical Center, MA.; Radiation Oncology (M.M.Q.), Boston Medical Center, MA.; Neurology (D. Strbian, S.R.), Helsinki University Hospital, University of Helsinki, Finland.; Radiology (P.V., R. Lauha, K.L.), Helsinki University Hospital, University of Helsinki, Finland.; Neuroradiology (C.H., M.A.M., J.J.), Heidelberg University Hospital, Germany.; Neurology (P.A.R., S.N.), Heidelberg University Hospital, Germany.; Neurology, Hospital Universitario Vall d’Hebron, Barcelona, Spain (M.O.-G., M. Ribo, M. Requena).; Radiology, Basel University Hospital, University of Basel, Switzerland (M.P., A.N.).; Neurology (J.B.K, D.H.), University of Erlangen-Nuremberg, Germany.; Neuroradiology (D.C.M., T.E.), University of Erlangen-Nuremberg, Germany.; Neurology, Institute of Diagnostic and Interventional Radiology (M. Köhrmann, J.K.E.), University Hospital Essen, Germany.; Neuroradiology (C.D.), University Hospital Essen, Germany.; Neurology, UZ Leuven, Belgium (J.D., R. Lemmens, L.V.).; Laboratory for Neurobiology, KU Leuven, Belgium (J.D., R. Lemmens, L.V.).; Neurology, Rhode Island Hospital (S.Y., L.S.).; Neurology (V.P.), University Hospital Carl Gustav Carus, Technische Universität Dresden, Germany.; Neuroradiology (D.P.O.K.), University Hospital Carl Gustav Carus, Technische Universität Dresden, Germany.; Dresden University Stroke Center (V.P., D.P.O.K.), University Hospital Carl Gustav Carus, Technische Universität Dresden, Germany.; Institute of Diagnostic and Interventional Neuroradiology, University Hospital Bern Inselspital, University of Bern, Switzerland (J.K., A. Mujanovic, B.S.).; Neurology, Grady Memorial Hospital (D.C.H., M.H.M., H.A., L.S.V.).; Neuroradiology, Centro Hospitalar Universitario de Lisboa Central, Portugal (B.C., I.F.).; NOVA Medical School, Universidade Nova de Lisboa, Portugal (I.F.).; Neurology and Stroke Unit, Department of Neuroscience, Bufalini Hospital, Cesena, Italy (M. Romoli).; Neuroradiology, University Hospital ‘San Giovanni di Dio e Ruggi d’Aragona’, Salerno, Italy (F.D.).; Neurology, The First Affiliated Hospital of USTC, Hefei, China (W.H., C.Z.).; Neurosurgery, Mount Sinai Health System (S.M., J.T.F.).; Neurology, McGovern Medical School at UTHealth, TX (S.A.S., S.S.-M.).; Neurology (J.P.M.), Hospital de Egas Moniz, Centro Hospitalar Lisboa Ocidental, Portugal.; Neuroradiology (J.N.R.), Hospital de Egas Moniz, Centro Hospitalar Lisboa Ocidental, Portugal.; Neuroradiology (M. Miszczuk, E.S.), Charité–Universitätsmedizin Berlin, Freie Universität Berlin and Humboldt-Universität zu Berlin, Germany.; Departement of Neurology and Experimental Neurology, and Center for Stroke Research Berlin (C.R., C.H.N.), Charité–Universitätsmedizin Berlin, Freie Universität Berlin and Humboldt-Universität zu Berlin, Germany.; Department of Neurology and Stroke (S.P., K.P.), University of Tübingen, Germany.; Hertie Institute for Clinical Brain Research (S.P., K.P.), University of Tübingen, Germany.; Neurosurgery, Barrow Neurological Institute, Phoenix, AZ (A.P.J., S.M.D.).; Radiology, Neuroradiology, and Nuclear Medicine, University Hospital Knappschaftskrankenhaus Bochum, Germany (V.M., M.K.).; Institute of Radiology and Neuroradiology, Klinikum Aschaffenburg, Germany (V.M.).; Neurosurgery, University of Buffalo, NY (A.H.S., A. Monteiro).; Neurology, New York Upstate Medical University (H.E.M.).; Neurology, University of Pittsburgh Medical Center, PA (N.S., R.G.N.).; Neurosurgery, University of South Florida, Tampa (M. Mokin, S.T.).; Radiology (K.A.), Turku University Hospital, Finland.; Neurology (P.Y.), Turku University Hospital, Finland.; Neurology, University of Chicago, IL (J.E.S.).; Miami Neuroscience Institute, FL (I.L., G.D.).; Neurology, University of Miami Miller School of Medicine, FL (N.A., V.S.).; Department of Neurology, University Hospital and University of Bern, Switzerland (U.F.).; Department of Neurology, University Hospital and University of Basel, Switzerland (U.F.).; Neuroradiology, University Hospital RWTH Aachen, Germany (C.S.W.).; Diagnostic and Interventional Neuroradiology, University Medical Center Hamburg-Eppendorf, Germany (U.H., L.M.).; Neurology, Klinikum Ludwigshafen, Germany (A.B., S.N.).

**Keywords:** case-control studies, functional status, posterior cerebral artery, stroke, thrombectomy

## Abstract

**BACKGROUND:**

Acute ischemic stroke with isolated posterior cerebral artery occlusion (iPCAO) lacks management evidence from randomized trials. We aimed to evaluate whether the association between endovascular treatment (EVT) and outcomes in iPCAO acute ischemic stroke is modified by initial stroke severity (baseline National Institutes of Health Stroke Scale [NIHSS]) and arterial occlusion site.

**METHODS:**

Based on the multicenter, retrospective, case-control study of consecutive iPCAO acute ischemic stroke patients (PLATO study [Posterior Cerebral Artery Occlusion Stroke]), we assessed the heterogeneity of EVT outcomes compared with medical management (MM) for iPCAO, according to baseline NIHSS score (≤6 versus >6) and occlusion site (P1 versus P2), using multivariable regression modeling with interaction terms. The primary outcome was the favorable shift of 3-month modified Rankin Scale (mRS). Secondary outcomes included excellent outcome (mRS score 0–1), functional independence (mRS score 0–2), symptomatic intracranial hemorrhage, and mortality.

**RESULTS:**

From 1344 patients assessed for eligibility, 1059 were included (median age, 74 years; 43.7% women; 41.3% had intravenous thrombolysis): 364 receiving EVT and 695 receiving MM. Baseline stroke severity did not modify the association of EVT with 3-month mRS distribution (*P*_interaction_=0.312) but did with functional independence (*P*_interaction_=0.010), with a similar trend on excellent outcome (*P*_interaction_=0.069). EVT was associated with more favorable outcomes than MM in patients with baseline NIHSS score >6 (mRS score 0–1, 30.6% versus 17.7%; adjusted odds ratio [aOR], 2.01 [95% CI, 1.22–3.31]; mRS score 0 to 2, 46.1% versus 31.9%; aOR, 1.64 [95% CI, 1.08–2.51]) but not in those with NIHSS score ≤6 (mRS score 0–1, 43.8% versus 46.3%; aOR, 0.90 [95% CI, 0.49–1.64]; mRS score 0–2, 65.3% versus 74.3%; aOR, 0.55 [95% CI, 0.30–1.0]). EVT was associated with more symptomatic intracranial hemorrhage regardless of baseline NIHSS score (*P*_interaction_=0.467), while the mortality increase was more pronounced in patients with NIHSS score ≤6 (*P*_interaction_=0.044; NIHSS score ≤6: aOR, 7.95 [95% CI, 3.11–20.28]; NIHSS score >6: aOR, 1.98 [95% CI, 1.08–3.65]). Arterial occlusion site did not modify the association of EVT with outcomes compared with MM.

**CONCLUSIONS:**

Baseline clinical stroke severity, rather than the occlusion site, may be an important modifier of the association between EVT and outcomes in iPCAO. Only severely affected patients with iPCAO (NIHSS score >6) had more favorable disability outcomes with EVT than MM, despite increased mortality and symptomatic intracranial hemorrhage.

The optimal management of patients presenting with acute ischemic stroke (AIS) resulting from isolated posterior cerebral artery occlusion (iPCAO) remains uncertain. Although endovascular treatment (EVT) shows a higher likelihood of arterial recanalization,^1^ its net clinical benefit over medical management (MM) in this context has yet to be established. Current knowledge regarding the association of EVT with safety and clinical outcomes for iPCAO stems from several retrospective studies yielding heterogeneous results.^1–7^ Two study-level meta-analyses did not show significant differences in 3-month disability, rates of symptomatic intracranial hemorrhage (sICH), or mortality between iPCAO patients treated with EVT and those with MM.^8,9^ The largest patient-level analysis, involving 1023 iPCAO patients from the endovascular therapy of PLATO (Posterior Cerebral Artery Occlusion Stroke) collaboration, found that patients treated with EVT and MM had similar 3-month disability, without any significant difference on the distribution of the modified Rankin Scale (mRS). However, a higher proportion of patients treated with EVT had early neurological improvement and achieved excellent 3-month outcome (mRS score 0–1), yet with higher rates of sICH.^6^ These findings do not lead to definitive conclusions but suggest that the effect size of EVT in unselected AIS from posterior cerebral artery occlusion (PCAO) might be small or even absent. Furthermore, it remains unclear whether certain subgroups of PCAO patients, such as those defined by stroke severity or by the occluded arterial segment, might benefit or be harmed from EVT.

Such heterogeneity of EVT’s effect, contingent upon clinical stroke severity and the occluded arterial segment, exists in other AIS settings. For instance, in anterior circulation stroke, the efficacy of EVT is established for proximal arterial segment occlusions (internal carotid artery, M1 segment of the middle cerebral artery) but remains under investigation for more distal occlusions. Regarding basilar artery occlusion, the effect of EVT seems to vary with clinical stroke severity, as there are indications of its efficacy in patients with moderate-to-severe stroke, but evidence is less conclusive in those with mild deficits.^10–13^

In this secondary analysis of the PLATO cohort, our aim was to assess whether, in AIS from iPCAO, the association of EVT with 3-month disability and safety outcomes is modified by baseline stroke clinical severity (measured by National Institutes of Health Stroke Scale [NIHSS]) and the site of arterial occlusion within the posterior cerebral artery (PCA).

## METHODS

### Ethics

Ethics committee or local institutional review board approval was obtained from all sites. Patient written informed consent was waived due to the retrospective nature of this study with anonymized data. The lead authors (T.N.N., D.S., S.N.) and the statistician (M.M.Q.) had password-protected access to all data centralized in the Helsinki University research platform. This study was reported according to the Strengthening the Reporting of Observational Studies in Epidemiology guidelines. Anonymized data not published in this article will be made available on request by a qualified investigator.

### Study Population

The PLATO study (URL: https://www.clinicaltrials.gov; unique identifier: NCT05291637) was an international, multicenter, retrospective, case-control study of consecutive patients aged ≥18 years with iPCAO treated between January 1, 2015, and August 1, 2022. Since the main publication, the PLATO study expanded with an additional 4 sites, totaling 31 sites across 9 countries included in this analysis.

The inclusion criteria for this secondary analysis were as follows: (1) patients ≥18 years of age diagnosed with ischemic stroke resulting from an isolated, unilateral occlusion of nonfetal PCA in the P1, P2 segment of the artery; (2) patient presentation within 24 hours of symptom onset; and (3) prestroke mRS score of 0 to 3. Patients were excluded if there was concomitant basilar artery occlusion or multiple vessel occlusion outside the PCA territory. In addition, we excluded patients with missing data in the covariates used in the analyses.

Patients were divided based on the treatment received: EVT versus MM, with or without intravenous thrombolysis. The decision to treat patients with EVT or MM was made by the treating medical team according to local treatment recommendations and the technical feasibility of performing EVT.

### Data Collection

We collected baseline demographics, clinical presentation, imaging parameters, and clinical and safety outcomes as described in the primary publication.^6^ Outcome at 3 months was part of each site’s stroke registry. The 3-month mRS score was prospectively collected during the routine clinical examination in the outpatient clinic or by a structured telephone interview by site investigators or coordinators, who could have been unblinded to the treatment received.

### Outcome Variables

The primary outcome of this analysis was the distribution of the 3-month mRS. Secondary outcomes included 3-month functional independence (defined as an mRS score 0–2 or, in patients with prestroke mRS score >2, return to baseline mRS), 3-month excellent outcome (defined as an mRS score 0 to 1 or, in patients with prestroke mRS score >1, return to baseline mRS), early neurological improvement (NIHSS score improvement by ≥2 points at 24 hours or at hospital discharge),^1,6^ sICH (defined as local or remote parenchymal hemorrhage type 2, subarachnoid hemorrhage, or intraventricular hemorrhage, combined with a neurological deterioration of ≥4 points on the NIHSS from baseline or leading to death, both judged causative by the attending physician), and 3-month mortality.

### Subgroup Definition

The heterogeneity of the association between EVT and outcomes was assessed by evaluating subgroups based on baseline NIHSS score and occlusion site. Regarding baseline NIHSS score, we defined 2 subgroups by dichotomization of baseline NIHSS score as ≤6 and >6. The lead investigators chose the selection of the 6-point cutoff because it approximated the median of the overall cohort and given the absence of data on an NIHSS treatment threshold in the published literature. In addition, we performed the same interaction analysis using NIHSS as a continuous variable. As for the occlusion site, we defined 2 subgroups: patients with P1 segment occlusion and those with P2 segment occlusion. These were defined as follows: P1 segment, from the branching point of the distal basilar artery to the branching point of the posterior communicating artery; P2 segment, from the PCA branching point of the posterior communicating artery, coursing around the midbrain to the quadrigeminal cistern.^6^ The PCA was considered as fetal (and patients excluded from the study) if the P1 segment was absent or hypoplastic, and the P2 segment was supplied primarily by the posterior communicating artery supplied by the internal carotid artery. Imaging assessment was conducted following local protocols, and the site of the arterial occlusion was determined through computed tomography angiography or magnetic resonance angiography. Each center’s investigators evaluated the site of arterial occlusion according to these predefined criteria.

### Statistical Analysis

We present continuous variables as median values with interquartile ranges (IQRs) and categorical variables as absolute numbers and percentages. We compared baseline and outcome variables by treatment (MM and EVT), baseline NIHSS score (≤6 and >6), and occlusion site (P1 and P2 segment occlusions), using the Pearson χ^2^ test for categorical variables and Mann-Whitney *U* test for continuous variables, as appropriate.

To assess the association between EVT and each outcome, we used multivariable regression models entering as independent variables the type of treatment together with prespecified baseline clinical and radiological variables known to be associated with the outcome. The following covariates were considered for different analyses: age, sex, baseline NIHSS score, year of treatment, prestroke mRS, hypertension, diabetes, atrial fibrillation, treatment with intravenous thrombolysis, posterior circulation Acute Stroke Prognosis Early Computed Tomography Score (pc-ASPECTS), and occlusion location. To account for the evolution of revascularization treatment over time, we included the year of stroke in the statistical models. The exact model for each analysis is described in the figure and table footnotes.

For the primary ordinal outcome, we utilized PROC GENMOD in SAS 9.4, with the cumulative logit link function and multinomial distribution. For the secondary binary outcomes, logistic regression models with logit link function and binomial distribution specifications were used. Clustering by sites was accounted for using a generalized estimating equation approach. An independent correlation structure with the smallest quasi-likelihood independence criterion value was assumed for the within-site clustering. To assess for potential heterogeneity in EVT association with outcomes, based on baseline NIHSS score and occlusion site, we entered an interaction term between the treatment variable and the subgroup variable in the regression models for each outcome. *P* values of the interaction term (*P* for interaction) were calculated.

The analyses were repeated using the propensity score–based inverse probability of treatment weighting (IPTW) method as an alternative model correcting for the same covariates. Using a multivariable logistic regression model, we first estimated the probability of EVT assignment (propensity score) conditional on the above covariates. For IPTW, the EVT group received weights of 1/propensity score, and MM received weights of 1/(1−propensity score). The weights for the EVT and MM groups were stabilized by replacing the numerator 1 with the proportion of patients receiving EVT and MM, respectively. These weights were then used in the regression models with interaction terms described above. All associations between predictors and outcomes are expressed as odds ratios and 95% CIs. All patients included in the above analyses had complete data on the covariates and the outcome variable included in the models. In instances of missing outcome data, we limited the analysis to patients with complete outcome data and reported the relative numbers. All tests were 2 sided, and *P* values <0.05 were considered significant. No adjustment for multiple testing was performed. We performed statistical analysis with SAS 9.4 (SAS Institute, Cary, NC) and R statistical software, version 4.3.1 (R Core Team 2023, R: A Language and Environment for Statistical Computing; R Foundation for Statistical Computing, Vienna, Austria).

## RESULTS

Of 1344 patients assessed for eligibility in the PLATO cohort, 239 did not meet the study inclusion criteria, and 46 were excluded because of missing covariate data, leaving 1059 patients for the current study (patient’s selection process in Figure S1; excluded patients had similar characteristics as patients included in the study, as shown in Table S1). The median (IQR) age of the study cohort was 74 (64–82) years, and 42.7% were women. Overall, 695 patients were treated with MM and 364 with EVT, and ≈40% of patients were treated with intravenous thrombolysis in both groups. Median NIHSS score was 3 points lower in the MM than in the EVT group (5 versus 8 points, respectively; *P*<0.001). Baseline clinical and radiological features and outcomes of the overall cohort, the EVT and MM groups, are displayed in Table S2. Of the 1059 patients initially included, 100 (9.4%) had missing data on the 3-month mRS and were, therefore, excluded from analyses on the primary outcome, excellent outcome, and functional independence. The number of patients with missing information for each outcome, along with those included in each respective analysis, is detailed in Figure S1.

**Figure 1.**
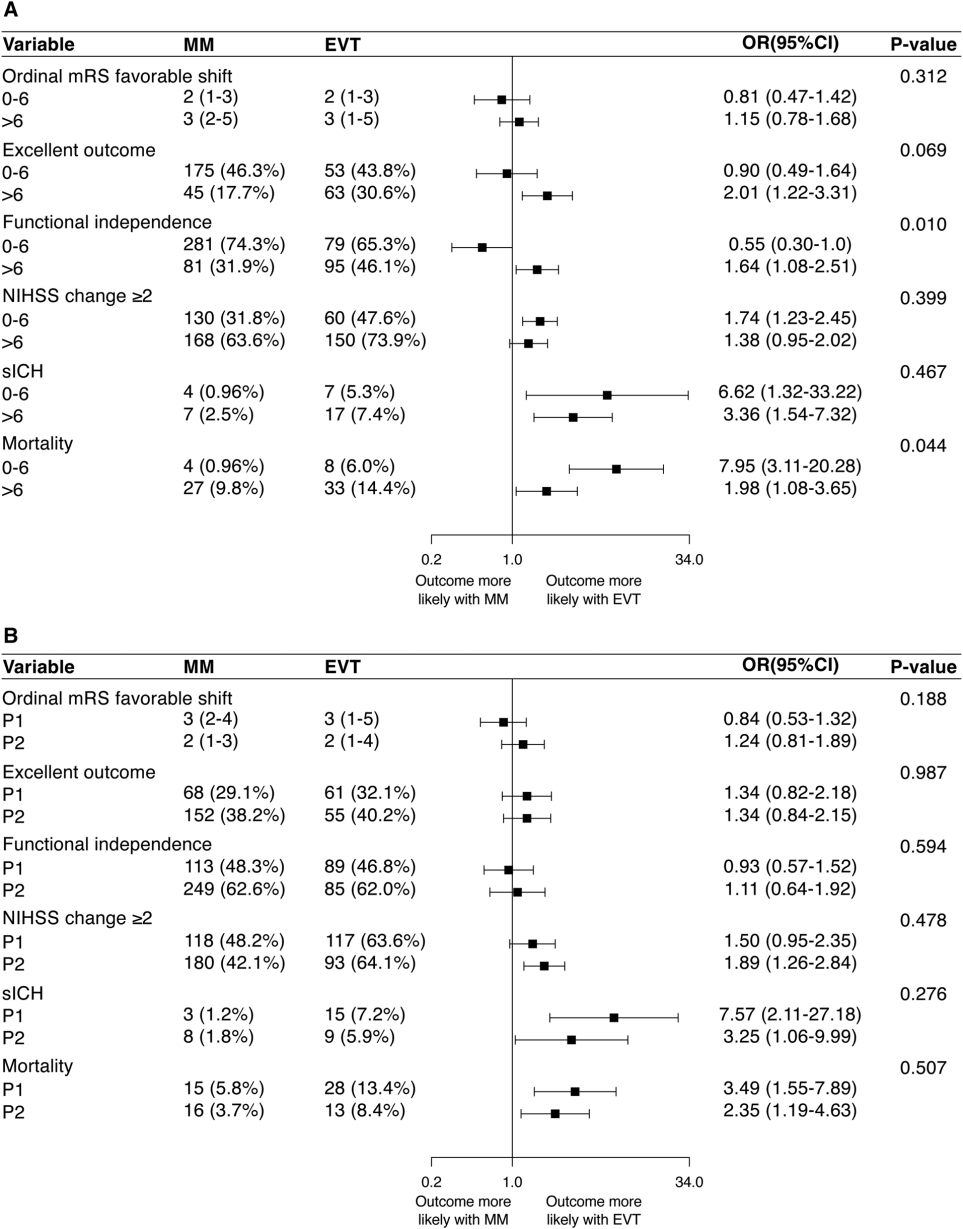
**Forest plot summarizing the odds ratio (OR) and their 95% CIs obtained from multivariable regression analysis for comparison of endovascular treatment (EVT) and medical management (MM) on ordinal modified Rankin Scale (mRS) favorable shift, excellent outcome, functional independence, National Institutes of Health Stroke Scale (NIHSS) decrease ≥2 points, symptomatic intracranial hemorrhage (sICH), and mortality.** Stratified by (**A**) baseline NIHSS score (0–6 vs >6) and (**B**) posterior cerebral artery segment occluded (P1 vs P2). The regression models were adjusted for the following covariates, in addition to baseline NIHSS score, segment occluded, and treated: age, sex, baseline mRS, year of treatment, posterior circulation Acute Stroke Prognosis Early Computed Tomography Score, hypertension, atrial fibrillation, diabetes, and intravenous tissue-type plasminogen activator.

**Figure 2.**
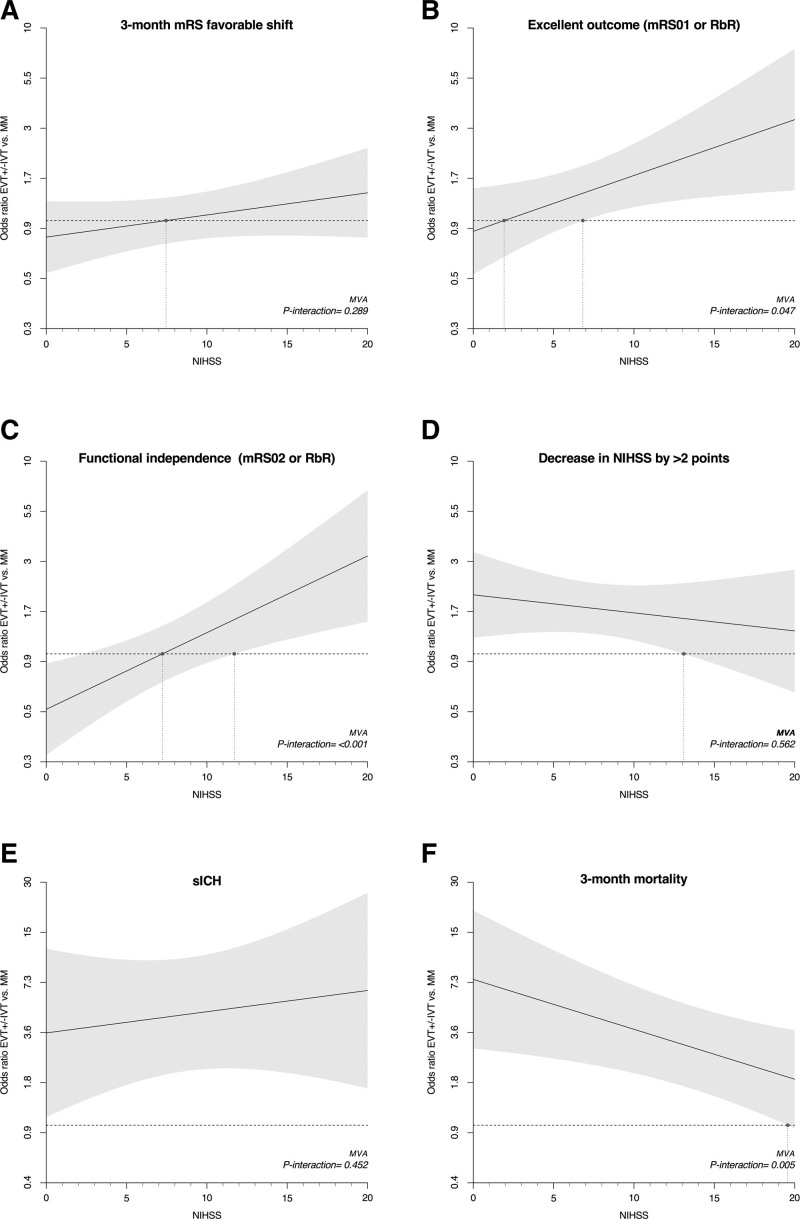
**Association of endovascular treatment (EVT) vs medical management (MM) and outcomes expressed as odds ratio (OR) and their 95% CIs, depending on continuous baseline National Institutes of Health Stroke Scale (NIHSS) score.** An OR >1 indicates that the outcome is more likely with EVT, whereas for OR <1, the outcome is more likely with MM. The outcomes displayed are (**A**) ordinal modified Rankin Scale (mRS) favorable shift, (**B**) excellent outcome, (**C**) functional independence, (**D**) NIHSS decrease ≥2 points, (**E**) symptomatic intracranial hemorrhage (sICH), and (**F**) mortality. The continuous dark line indicates the point estimate of the OR associated with EVT vs MM at each NIHSS value. The shaded gray area reflects the 95% CI of the odds ratio. The vertical dotted lines indicate the NIHSS value at which the point estimate and the lower bound of the 95% CI crosses 1. ORs are obtained from multivariable regression analysis (MVA) with interaction term between treatment and baseline NIHSS score. Models were adjusted for age, sex, baseline mRS, year of treatment, posterior circulation Acute Stroke Prognosis Early Computed Tomography Score, hypertension, atrial fibrillation, diabetes, and intravenous tissue-type plasminogen activator and segment occluded. IVT indicates intravenous thrombolysis; and RbR, return to baseline Rankin.

Patients with baseline NIHSS score >6, compared with those with NIHSS score ≤6, were older (median [IQR], 76 [67–83] versus 72 [61–80]; *P*<0.0001), more frequently female (46.9% versus 38.8%; *P*=0.009), had higher prestroke disability, had more visual field defect (74.2% versus 65.6%; *P*=0.004), had more vascular risk factors, and had higher frequency of P1 occlusion (54.9% versus 34.5%; *P*<0.0001). They were more frequently treated with EVT (45.5% versus 24.1%; *P*<0.0001), and EVTs were more often performed within 6 hours (72.4% versus 65.6%; *P*=0.027; Table 1). Patients with baseline NIHSS score >6 had higher likelihood of early neurological improvement (NIHSS score change ≥2: MVA-adjusted odds ratio [aOR], 4.24 [95% CI, 3.22–5.58]) but less favorable 3-month disability outcomes (3-month mRS favorable shift: MVA-aOR, 0.33 [95% CI, 0.27–0.41]) and higher mortality (MVA-aOR, 3.93 [95% CI, 2.05–7.52]) compared with patients with baseline NIHSS score of 0 to 6 (Table 2). The interaction analysis between baseline NIHSS score and EVT showed that the baseline NIHSS score did not modify the association of EVT with the distribution of 3-month mRS (*P*_interaction_=0.312). However, baseline NIHSS score modified the association of EVT with functional independence (*P*_interaction_=0.010) and mortality (*P*_interaction_=0.044), and a similar yet nonsignificant trend was observed for excellent outcome (*P*_interaction_=0.069; Figure 1A and Table S3 for IPTW analysis). Specifically, EVT was associated with more excellent outcome and functional independence than MM in patients with baseline NIHSS score >6 (mRS score 0–1: 30.6% versus 17.7%; aOR, 2.01 [95% CI, 1.22–3.31]; mRS score 0–2: 46.1% versus 31.9%; aOR, 1.64 [95% CI, 1.08–2.51]), while in patients with baseline NIHSS score ≤6, the difference was absent or less favorable for EVT (mRS score 0–1: 43.8% versus 46.3%; aOR, 0.90 [95% CI, 0.49–1.64]; mRS score 0–2: 65.3% versus 74.3%; aOR, 0.55 [95% CI, 0.30–1.0]). SICH and mortality were higher in patients treated with EVT compared with those receiving MM, regardless of baseline NIHSS score being ≤6 or >6 (*P*_interaction_=0.467), but the mortality increase was significantly more pronounced in the former group (*P*_interaction_=0.044; NIHSS score ≤6: aOR, 7.95 [95% CI, 3.11–20.28]; NIHSS score >6: aOR, 1.98 [95% CI, 1.08–3.65]). Similarly, we detected an interaction between EVT and NIHSS as a continuous variable for excellent outcome (*P*_interaction_=0.047), functional independence (*P*_interaction_<0.001), and mortality (*P*_interaction_=0.005; Figure 2A through 2F and Figure S2A through S2F for IPTW analysis). In the multivariable analysis, EVT was associated with more excellent outcome and functional independence starting from baseline NIHSS scores >6 and >11, respectively (as indicated by the point at which the lower boundary of the 95% CI of the odds ratio crossed 1, indicating no association; Figure 2B and 2C). Similar results were obtained in the IPTW analysis, where the association of EVT with excellent outcome and functional independence was present starting from baseline NIHSS scores >4 and >8, respectively (Figure S2B and S2C).

**Table 1.**
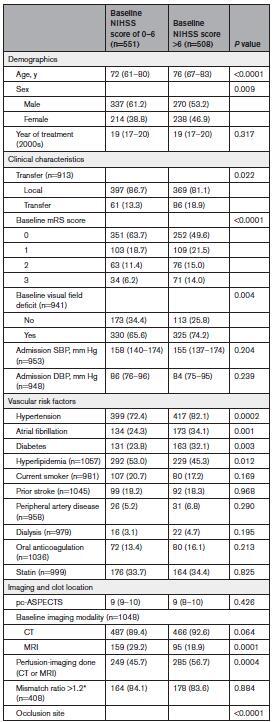

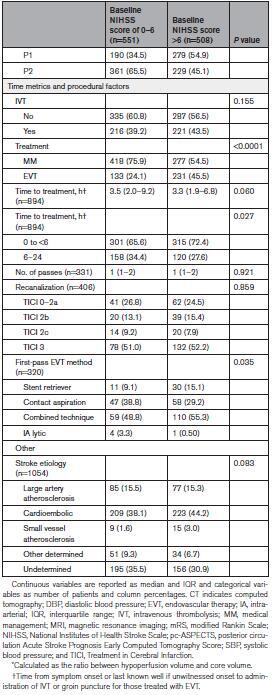
Baseline Characteristics and Metrics of Patients With Posterior Cerebral Artery Occlusion by Baseline NIHSS Score

**Table 2.**
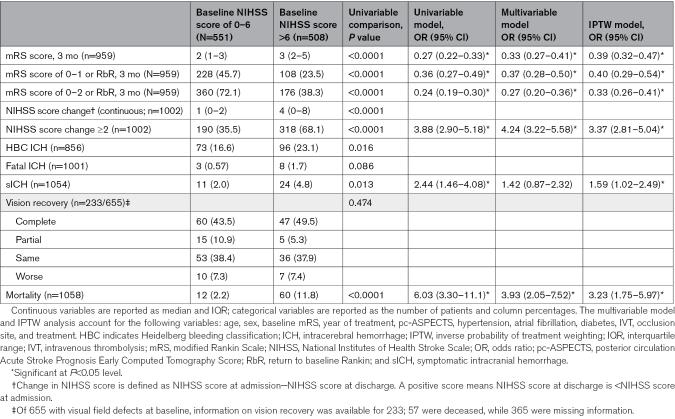
Outcomes of Patients With Posterior Cerebral Artery Occlusion by Baseline NIHSS Score

When comparing patients with occlusions in the P1 and P2 segments of the PCA, those with P1 segment occlusion presented with higher baseline NIHSS score (median [IQR], 8 [4–13] versus 5 [3–8]; *P*<0.0001), less frequent visual field deficits (63.9% versus 74.1%; *P*=0.001), and lower baseline pc-ASPECTS (median [IQR], 9 [8–10] versus 9 [9–10]; *P*=0.004; Table S4). They had similar rates of intravenous thrombolysis administration (38.4% versus 43.6%; *P*=0.089) but underwent EVT more frequently (44.8% versus 25.6%; *P*<0.001). Procedural metrics among EVT-treated patients were comparable between patients with P1 and P2 occlusions. While univariable analysis showed that P2 occlusion was associated with a more favorable mRS distribution, a higher proportion of excellent outcome and functional independence, and lower mortality, these differences were not significant when adjusting for potential confounders in multivariable and IPTW analyses (Table 3). We did not detect an interaction between EVT and the occluded arterial segment with regard to any of these outcomes. EVT was associated with a higher likelihood of early neurological improvement but also higher rates of sICH and mortality without a significant association with the overall 3-month mRS distribution. These findings were consistent across the entire cohort and within the P1 and P2 occlusions both in multivariable and IPTW analyses (Figure 1B; Table S5).

**Table 3.**
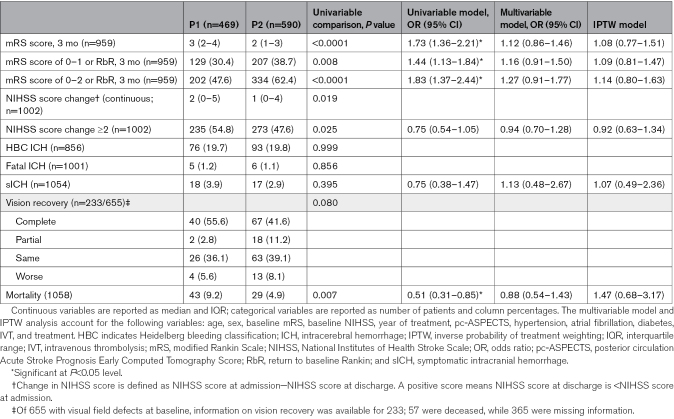
Outcomes of Patients With Posterior Cerebral Artery Occlusion by Segment of Arterial Occlusion

## DISCUSSION

This secondary analysis of the international multicenter PLATO cohort demonstrated that among patients presenting with iPCAO within 24 hours of symptom onset, baseline NIHSS score significantly modified the association of EVT with outcomes. In moderate-to-severe iPCAO stroke, EVT was associated with a higher rate of excellent outcome and functional independence, despite higher mortality and sICH. In mild iPCAO stroke, the increased mortality associated with EVT compared with MM was more pronounced, sICH was similarly increased, and the difference on disability outcomes was absent or less in favor of EVT. The site of arterial occlusion in P1 or P2 segments of the PCA did not modify the association of EVT with any of the clinical or safety outcomes.

According to our findings, the initial stroke severity measured by NIHSS emerges as an important parameter in guiding EVT selection for iPCAO patients. In patients with mild deficits at baseline, the potential advantages of arterial recanalization with EVT may be outweighed by procedural complications and a higher risk of sICH. Indeed, these complications usually yield a pronounced impact on outcomes,^14^ which is likely to weigh even more in the population with baseline mild deficits. Conversely, in patients presenting with more severe strokes, the advantage of revascularization may balance or outweigh the risks associated with EVT, and the impact of procedural complications might be comparatively less significant. Our analysis of baseline NIHSS score as a continuous variable provides results consistent with those from the dichotomized NIHSS approach. It also suggests that the NIHSS threshold at which the treatment-outcome association becomes evident varies depending on the outcome measure. For instance, the outcome association with EVT compared with MM became apparent at lower NIHSS values for the mRS score 0 to 1 end point, whereas a higher NIHSS threshold was required to show better EVT outcomes when mRS score 0 to 2 was used as an outcome measure. In other words, patients with a lower baseline NIHSS score may have less likelihood of showing EVT benefit compared with MM on higher Rankin disability end points, such as mRS scale 0 to 2. Our findings on the role of baseline NIHSS score are rather novel, as previous observational studies on iPCAO either did not perform subgroup analysis evaluating 3-month outcome as stratified by baseline NIHSS score^3,7^ or did not detect better 3-month outcomes (mRS scores 0–1 and 0–2) with EVT compared with MM in patients with NIHSS score >10, possibly due to lower sample size.^5^ Nevertheless, the TOPMOST (Treatment for Primary Medium Vessel Occlusion Stroke) and EaT-PeCANpIeS (Endovascular Thrombectomy for Posterior Cerebral Artery Strokes in the National Inpatient Sample) studies reported results on short-term outcome consistent with ours,^3,7^ revealing a greater probability of neurological improvement and better mRS at hospital discharge with EVT in iPCAO patients with more severe stroke. Similar trends regarding the influence of baseline NIHSS score have been observed in studies looking at other intracranial occlusions, particularly in basilar artery occlusions, where potential benefits have been suggested in patients with baseline NIHSS score ≥6, whereas outcomes are less certain for those with mild clinical stroke severity.^10,11,13^ Similarly, in anterior circulation occlusion, the benefit of EVT has been established primarily for AIS with moderate-to-severe strokes, while its efficacy in patients presenting with mild deficits remains under debate.^15,16^ Notably, anterior circulation LVOs with initial minor deficit not receiving EVT often result in early neurological deterioration, significantly impacting medium-term outcomes. This deterioration is commonly attributed to a breakdown of collateral supply and is potentially preventable by immediate EVT.^17^ Interestingly, PCAOs receiving MM appear to have a lower rate of early neurological deterioration,^5,17^ which could further diminish the potential benefit of EVT.

The absence of influence of arterial occlusion site on EVT outcomes might appear unexpected but has already been reported in previous studies.^3–5^ This probably indicates that the distinction between the P1 and P2 segments may not comprehensively capture the different aspects of PCAO. Indeed, distal P1 occlusions could resemble proximal P2 occlusions in many aspects, such as functional impact, stroke clinical severity, and technical accessibility. Conversely, within the P1 segment, occlusions might exhibit substantial heterogeneity depending on the involvement of thalamic or mesencephalic structures, mesiotemporal areas, the visual cortex, and the presence (or absence) of the posterior communicating artery supply of the P2 segment. Similarly, P2 occlusions may significantly vary in functional impact, depending on the involvement of the visual cortex and accessibility to EVT, which pivots on whether the occlusion is more proximal or distal within the P2 segment.^18^ Consequently, owing to the similarities between the P1 and P2 occlusions and the clinical heterogeneity within each segment, the P1 versus P2 distinction might bear less relevance in the AIS setting and the selection of acute revascularization treatments. As an alternative explanation, our adjustment of this analysis with baseline NIHSS score could have eliminated a possible effect of EVT on occlusion location.

Our study has limitations. First, the observational design, where patient allocation to treatment groups relied on the discretion of treating physicians, incurs selection bias. Second, patients were included from routine clinical practice, potentially leading to reporting bias due to unblinded outcome assessment. Third, the EVT group presented with a more severe baseline neurological deficit, which we adjusted for by conducting multivariable and IPTW analyses. The congruence of results across these analyses reinforces the validity of our findings. Additionally, we acknowledge that the NIHSS might not be the most suitable scale for assessing deficits resulting from PCA territory infarction.^19,20^ Besides, while pc-ASPECTS was developed to quantify ischemic changes in the posterior circulation, that is, basilar artery occlusion,^21^ it might not best capture the extent of stroke in iPCAO. Therefore, it is not surprising that both treatment groups in our study had high median pc-ASPECTS. Also, no central reading to ascertain the site of occlusion or pc-ASPECTS score was performed. Furthermore, although our study documented follow-up NIHSS, 3-month mRS, sICH, and mortality, vision examination at 3 months was missing in a substantial proportion of patients with baseline visual field defects. Consequently, we could not perform an analysis of this outcome. Finally, we did not evaluate cognitive outcomes, which may be a clinically meaningful consequence of PCAO.^4^

In conclusion, our findings suggest that baseline NIHSS score, rather than the site of arterial occlusion, appears to be an important modifier of the association of EVT versus MM and outcomes in iPCAO. EVT was associated with more favorable disability outcomes than MM in moderate-to-severe iPCAO strokes, while this difference was absent or less in favor of EVT in minor strokes. Mortality was higher with EVT compared with MM in both minor and severe stroke but to a greater extent in the latter group. sICH was increased with EVT irrespectively of baseline NIHSS score. These observations might be important for hypothesis generation in future randomized studies exploring the efficacy of EVT for iPCAO.

## ARTICLE INFORMATION

### Authors

Davide Strambo, MD; Patrik Michel, MD; Thanh N. Nguyen, MD; Mohamad Abdalkader, MD; Muhammad M. Qureshi, MBBS, MPH; Daniel Strbian, MD, PhD, MSc; Christian Herweh, MD; Markus A. Möhlenbruch, MD; Silja Räty, MD, PhD; Marta Olive-Gadea, MD; Marc Ribo, MD, PhD; Marios Psychogios, MD; Urs Fischer, MD, MS; Anh Nguyen, MD; Joji B. Kuramatsu, MD; David Haupenthal, MD; Martin Köhrmann, MD; Cornelius Deuschl, MD; Jordi Kühne Escolà, MD; Jelle Demeestere, MD, PhD; Robin Lemmens, MD, PhD; Lieselotte Vandewalle, MD; Shadi Yaghi, MD; Liqi Shu, MD; Volker Puetz, MD; Daniel P.O. Kaiser, MD; Johannes Kaesmacher, MD, PhD; Adnan Mujanovic, MD; Dominique Cornelius Marterstock, MD; Tobias Engelhorn, MD; Manuel Requena, MD; Hormuzdiyar H. Dasenbrock, MD, MPH; Piers Klein, MA; Diogo C. Haussen, MD; Mahmoud H. Mohammaden, MD; Hend Abdelhamid, MD; Lorena Souza Viana, MD; Bruno Cunha, MD; Isabel Fragata, MD, MSc, PhD; Michele Romoli, MD, PhD; Francesco Diana, MD; Wei Hu, MD, PhD; Chao Zhang, MD; Pekka Virtanen, MD; Riikka Lauha, MD; Jessica Jesser, MD; Judith Clark, RN; Stavros Matsoukas, MD; Johanna T. Fifi, MD; Sunil A. Sheth, MD; Sergio Salazar-Marioni, MD; João Pedro Marto, MD; João Nuno Ramos, MD; Milena Miszczuk, MD; Christoph Riegler, MD; Sven Poli, MD, MSc; Khouloud Poli, MD; Ashutosh P. Jadhav, MD, PhD; Shashvat M. Desai, MD; Volker Maus, MD; Maximilian Kaeder, MD; Adnan H. Siddiqui, MD, PhD; Andre Monteiro, MD; Hesham E. Masoud, MD; Neil Suryadareva, MD; Maxim Mokin, MD, PhD; Shail Thanki, MD; Kemal Alpay, MD, PhD; Pauli Ylikotila, MD, MSc; James E. Siegler, MD; Italo Linfante, MD; Guilherme Dabus, MD; Negar Asdaghi, MD; Vasu Saini, MD; Christian H. Nolte, MD; Eberhard Siebert, MD; Bettina L. Serrallach, MD; Charlotte S. Weyland, MD; Uta Hanning, MD; Lukas Meyer, MD; Anne Berberich, MD; Peter A. Ringleb, MD; Raul G. Nogueira, MD; Simon Nagel, MD

### Sources of Funding

None.

### Disclosures

Dr Dabus: consultancy for Cerenovus, Penumbra, Route 92, Medtronic, MicroVention, and Stryker; stock holdings in RIST and InNeuroCo. Dr Fifi: consultancy for Cerenovus, MicroVention, and Stryker; Data Safety Monitoring Board (DSMB) for MIVI; stock holdings in Imperative Care and Sim&Cure. Dr Fischer: research support from the Swiss National Science Foundation (SNF), Medtronic, Stryker, Rapid Medical, Penumbra, and Phenox; consultancies for Stryker and CSL Behring; is on the advisory board for Alexion/Portola, Boehringer Ingelheim, Biogen, and Acthera. Dr Haussen: consultancy for Vesalio, Cerenovus, Stryker, Brainomix, Poseydon Medical, and Chiesi USA; DSMB for Jacobs Institute; stock options in viz AI. Dr Herweh: consultancy for Brainomix; speaker with Stryker. Dr Jadhav: consulting with Basking Biosciences; stock options in Gravity Medical Technology; a patent for a novel stent retriever device licensed to Basking Biosciences; and Editor-in-Chief for the *Stroke: Vascular and Interventional Neurology* journal. Dr Kaesmacher: grants from the Swiss Academy of Medical Sciences/Bangerter Foundation, Swiss Stroke Society, and Clinical Trials Unit Bern. Dr Kaiser: grants from the Joachim Herz Foundation. Dr Kuramatsu: grants from Alexion Pharmaceuticals, Bayer Healthcare, Sanofi Pasteur, and Biogen Idec. Dr Marto: consulting and speaker fees from Amicus Therapeutics and Boehringer Ingelheim. Dr Michel: grants from the University of Lausanne and SNF. Dr Möhlenbruch: grants from Medtronic, Stryker, and MicroVention. Dr Mokin: stock holdings in BrainQ, Serenity Medical, Synchron, and Bendit Technology; consulting at MicroVention, Medtronic, and Johnson & Johnson. Dr Nagel: consultancy for Brainomix; speaker at Boehringer Ingelheim and Pfizer. Dr Nguyen: Associate Editor of *Stroke*, advisory board at Aruna Bio and Brainomix. Dr Nogueira: consultancy for Biogen, Brainomix, Corindus, Cerenovus, Stryker, Medtronic, Ceretrieve, Anaconda Biomed, Vesalio, Imperative Care, NeuroVasc Technologies, viz AI, Genentech, Prolong Pharmaceuticals, Perfuze, Phenox, and RapidPulse; stock options in viz AI, Vesalio, Perfuze, Corindus, Brainomix, and Ceretrieve; grants from Cerenovus and Stryker. Dr Nolte: research support and compensation from Novartis, AstraZeneca, Deutsches Zentrum für Herz-Kreislaufforschung, and Deutsches Zentrum für Neurodegenerative Erkrankungen; consultancy for Alexion, Daiichi Sankyo, Novartis, AstraZeneca, Bayer Healthcare, Pfizer, Alexion, and Bristol Myers Squibb. S. Poli: research grants from BMS/Pfizer, Boehringer Ingelheim, Daiichi Sankyo, German Federal Joint Committee Innovation Fund, and German Federal Ministry of Education and Research, Helena Laboratories and Werfen as well as speakers’ honoraria/consulting fees from Alexion, AstraZeneca, Bayer, Boehringer Ingelheim, Bristol Myers Squibb/Pfizer, Daiichi Sankyo, Portola, and Werfen (all outside of the submitted work). Dr Psychogios: grants from Penumbra, Rapid Medical, Medtronic, Phenox, Bangerter-Rhyner Stiftung, SNF, Siemens Healthineers, and Stryker Neurovascular; travel support from Medtronic, Siemens Healthineers, Phenox, Penumbra, and Stryker; consultancy for Siemens Healthineers. Dr Puetz: lecturer for Daiichi Sankyo. Dr Ribo: consultancy for Medtronic MiniMed, Cerenovus, AptaTargets, Stryker, and Philips; stock holdings in Methinks, Nora, and Anaconda Biomed. Dr Ringleb: travel support from Bayer and Bristol Myers Squibb; consultancy for Daiichi Sankyo Company and Boehringer Ingelheim. Dr Romoli: research grants from the Italian Stroke Association; consultancy for CSL Behring. Dr Sheth: consultancy for Imperative Care, viz AI, and Penumbra; compensation from Motif Neurosciences (other services); grants from the National Institutes of Health. Dr Siddiqui: ownership stake in Integra Lifesciences and Medtronic; consultancy for Cordis, Rapid Medical, MicroVention, Medtronic Vascular, Vassol, IRRAS USA, Boston Scientific, Amnis Therapeutics, Minnetronix Neuro, Canon Medical Systems USA, Cardinal Health 200, Johnson & Johnson–Latin America, Corindus, Penumbra, Apellis Pharmaceuticals, W.L. Gore & Associates, Stryker Corporation, and viz AI; stock holdings in E8, Spinnaker Medical, Endostream Medical, Cerebrotech Medical Systems, Adona Medical, Bend IT Technologies, Whisper Medical, Neurotechnology Investors, Collavidence, Instylla, Q’Appel Medical, Serenity Medical, Borvo Medical, NeuroRadial Technologies, Sense Diagnostics, Tulavi Therapeutics, Synchron, Neurolutions, Viseon, BlinkTBI, Radical Catheter Technologies, and Truvic Medical; stock options in viz AI, StimMed, Three Rivers Medical, Silk Road Medical, Imperative Care, CVAid Ltd, Cerevatech Medical, InspireMD, PerFlow Medical; security holdings in Vastrax, Launch NY, QAS.ai, VICIS, Inc, Neurovascular Diagnostics, Cognition Medical, and SongBird Therapy. Dr Strbian: Assistant Editor of *Stroke*, Editorial Board of *European Stroke Journal*, advisory board at Boehringer Ingelheim, Alexion/AstraZeneca, and Bristol Myers Squibb/Janssen; research support from Boehringer Ingelheim; consultancies for Orion, Herantis Pharma, and CSL Behring. The other authors report no conflicts.

### Supplemental Material

Tables S1–S5

Figures S1–S2

## Supplementary Material


