# Supplementary material for: Endovascular Versus Medical Therapy in Posterior Cerebral Artery Stroke: Role of Baseline NIHSS Score and Occlusion Site
Source: Stroke. 2024 May 16;55(7):1787–97. doi: 10.1161/STROKEAHA.124.047383 (PMC11198954; doi:10.1161/STROKEAHA.124.047383)

Endovascular versus medical therapy in posterior cerebral artery stroke: role of baseline NIHSS and occlusion site

SUPPLEMENTARY MATERIAL

Supplementary figures legend

Figure S1. Patient's selection process applying inclusion and exclusion criteria of the study. mRS indicates modified Rankin Scale; NIHSS, National Institutes of Health Stroke Scale; PCA, posterior cerebral artery; pc-ASPECTS, posterior circulation Acute Stroke Prognosis Early CT Score; RbR, return to baseline Rankin score.

Figure S2. Association of endovascular treatment (EVT) versus medical management (MM) and outcomes expressed as odds ratio (OR) and their 95% confidence intervals (CI), depending on continuous baseline NIHSS. An OR greater than 1 indicates that the outcome is more likely with EVT, whereas for OR<1 the outcome is more likely with MM. The outcomes displayed are: (A) ordinal modified Rankin Scale (mRS) favorable shift, (B) excellent outcome, (C) functional independence, (D) NIHSS decrease ≥ 2 points, (E) symptomatic intracranial hemorrhage (sICH), and (F) mortality. The continuous dark line indicates the point estimate of the OR associated with EVT vs MM at each NIHSS value. The shaded gray area reflects the 95% CI of the odds ratio. The vertical dotted lines indicate the NIHSS value at which the point estimate and the lower bound of the 95% CI crosses 1 (displayed only in case of statistically significant interaction between baseline NIHSS and treatment). ORs are obtained from regression models with interaction term between treatment and baseline NIHSS, using inverse probability of treatment weighting (IPTW) to account for the following covariates: age, sex, baseline mRS, year of treatment, pc-ASPECTS, hypertension, atrial fibrillation, diabetes mellitus, IV tPA, and segment occluded.

Table S1. Baseline characteristics and metrics of patients of the overall cohort, those included and excluded in the study.

	Overall (n=1344)	Included (N=1059)	Excluded (N=285)	*P*
Demographics				
Age, years	74 (64-82)	74 (64-82)	75.5 (64-84)	0.156
Sex				0.903
Male	768 (57.2)	607 (57.3)	161 (56.7)	
Female	575 (42.8)	452 (42.7)	123 (43.3)	
Year of treatment (2000s)	19 (17-20)	19 (17-20)	19 (17.7-20)	0.244
Clinical characteristics				
Transfer				0.511
Local	936 (83.5)	766 (83.9)	170 (81.7)	
Transfer	185 (16.5)	147 (16.1)	38 (18.3)	
Baseline mRS				<0.001
0	733 (55.5)	603 (56.9)	130 (49.8)	
1	252 (19.1)	212 (20)	40 (15.3)	
2	156 (11.8)	139 (13.1)	17 (6.5)	
3	126 (9.5)	105 (9.9)	21 (8.1)	
4	44 (3.3)	0 (0)	44 (16.9)	
5	9 (0.7)	0 (0)	9 (3.5)	
Baseline NIHSS (continuous)	6 (3-11)	6 (3-10)	5 (2-13)	0.137
Baseline NIHSS (strata)				<0.001
0-6	709 (52.8)	551 (52)	158 (55.6)	
7-19	516 (38.4)	429 (40.5)	87 (30.6)	
≥20	118 (8.8)	79 (7.5)	39 (13.7)	
Baseline visual field deficit				0.912
No	349 (30.3)	286 (30.4)	63 (29.7)	
Yes	804 (69.7)	655 (69.6)	149 (70.3)	
SBP, mmHg	157 (139-173.1)	157 (139-174)	158 (138.7-172)	0.716
DBP, mmHg	84 (75-95)	85 (75-95)	81 (71-93)	0.002
Vascular risk factors				
Hypertension	1037 (77.4)	816 (77)	221 (78.7)	0.626
Atrial fibrillation	401 (30)	307 (29)	94 (33.7)	0.147
Diabetes mellitus	377 (28.1)	294 (27.8)	83 (29.5)	0.607
Hyperlipidemia	664 (49.6)	521 (49.3)	143 (50.9)	0.682
Current Smoker	225 (18.5)	187 (19.1)	38 (16.1)	0.338
Prior Stroke	259 (19.8)	191 (18.3)	68 (25.6)	0.010
Peripheral Artery Disease	79 (6.5)	57 (6)	22 (8.5)	0.183
Dialysis	46 (3.8)	38 (3.9)	8 (3.6)	0.999
Oral anticoagulation	191 (14.7)	152 (14.7)	39 (14.9)	0.990
Statin	433 (34.8)	340 (34)	93 (37.8)	0.299

Table S1. Baseline characteristics and metrics of patients of the overall cohort, those included and excluded in the study.

	Overall (n=1344)	Included (N=1059)	Excluded (N=285)	*P*
Imaging and clot location				
pc-ASPECTS	9 (9-10)	9 (9-10)	9 (8-10)	0.011
Baseline imaging modality				
CT	1170 (89.7)	953 (90.9)	217 (84.8)	0.005
MRI	328 (25.2)	254 (24.2)	74 (29)	0.134
Perfusion-imaging done (CT or MRI)	655 (50.2)	534 (51)	121 (47.3)	0.323
Mismatch ratio > 1.2[+]	401 (80.7)	342 (83.8)	59 (66.3)	<0.001
Occlusion site				<0.001
P1	518 (38.5)	469 (44.3)	49 (17.2)	
P2	660 (49.1)	590 (55.7)	70 (24.6)	
P3-4	127 (9.4)	0 (0)	127 (44.6)	
Bilateral occlusion	13 (1)	0 (0)	13 (4.6)	
Fetal PCA	18 (1.3)	0 (0)	18 (6.3)	
Multiple vessels	8 (0.6)	0 (0)	8 (2.8)	
Time metrics and procedural factors				
IV tPA				0.001
No	822 (61.2)	622 (58.7)	200 (70.2)	
Yes	522 (38.8)	437 (41.3)	85 (29.8)	
Treatment				0.002
MM	910 (67.7)	695 (65.6)	215 (75.4)	
EVT	434 (32.3)	364 (34.4)	70 (24.6)	
Time to treatment*, h	2.9 (1.8-5.0)	3.3 (2.0-7.7)	2.5 (1.7-5.0)	0.189
Time to treatment*, h				0.831
0 - <6	717 (70.3)	616 (68.9)	101 (80.2)	
6 – 24	303 (19.7)	278 (31.1)	25 (19.8)	
Number of passes	1 (1-2)	1 (1-2)	1 (1-2)	0.546
Recanalization				0.863
TICI 0-2a	93 (21.1)	77 (21.2)	21 (25.6)	
TICI 2b	69 (15.7)	55 (15.1)	14 (17.1)	
TICI 2c	39 (8.8)	34 (9.3)	5 (6.1)	
TICI 3	240 (54.4)	198 (54.4)	42 (51.2)	
First pass EVT method				0.387
Stent retriever	50 (13.3)	41 (12.8)	9 (15.8)	
Contact Aspiration	129 (34.2)	105 (32.8)	24 (42.1)	
Combined technique	192 (50.9)	169 (52.8)	23 (40.4)	
IA lytic	6 (1.6)	5 (1.6)	1 (1.8)	
Other				
Stroke etiology				0.214
Large artery atherosclerotic	201 (15)	162 (15.4)	39 (13.8)	
Cardioembolic	546 (40.8)	432 (41)	114 (40.3)	
Small vessel atherosclerotic	25 (1.9)	24 (2.3)	1 (0.3)	
Other determined	109 (8.2)	85 (8.1)	24 (8.5)	
Undetermined	456 (34.1)	351 (33.3)	105 (37.1)	

Continuous variables are reported as median and interquartile range, categorical variables are reported as number of patients and column percentages.

Abbreviations: MM, Medical Management; EVT, Endovascular Therapy; N, the total number of patients; mRS, modified Rankin Scale; NIHSS, National Institutes of Health Stroke Scale; SBP, systolic blood pressure; DBP, diastolic blood pressure; pc-ASPECTS, posterior circulation Acute Stroke Prognosis Early CT Score; CT, computed tomography; MRI, Magnetic resonance imaging; IV tPA, intravenous tissue-type plasminogen activator; TICI, Treatment in Cerebral Infarction; IA, intra-arterial.

[+]Calculated as the ratio between hypoperfusion volume and core volume.

*Time from symptom onset or last known well if unwitnessed onset, to administration of IVT or groin puncture for those treated with EVT.

Table S2A. Baseline characteristics, metrics, and outcomes of patients with P1 and P2 occlusion of posterior cerebral artery (PCA) occlusion treated with Medical Management (MM) vs. Endovascular thrombectomy (EVT).

	Overall (N=1,059)	MM (N=695)	EVT (N=364)	*P*
Demographics				
Age, years	74 (64-82)	74 (64-81)	74 (64-82)	0.917
Sex				0.634
Male	607 (57.3)	402 (57.8)	205 (56.3)	
Female	452 (42.7)	293 (42.2)	159 (43.7)	
Year of treatment (2000s)	19 (17-20)	18 (17-20)	19 (18-20)	<0.0001
Clinical characteristics				
Transfer (N=913)				<0.0001
Local	766 (83.9)	514 (88.6)	252 (75.7)	
Transfer	147 (16.1)	66 (11.4)	81 (24.3)	
Baseline mRS				0.008
0	603 (56.9)	373 (53.7)	230 (63.2)	
1	212 (20.0)	141 (20.3)	71 (19.5)	
2	139 (13.1)	103 (14.82)	36 (9.9)	
3	105 (9.9)	78 (11.2)	27 (7.4)	
Baseline NIHSS (continuous)	6 (3-10)	5 (3-10)	8 (5-12)	<0.0001
Baseline NIHSS (strata)				<0.0001
0-6	551 (52)	418 (60.1)	133 (36.5)	
7-19	429 (40.5)	229 (33)	200 (55)	
≥20	79 (7.5)	48 (6.9)	31 (8.5)	
Baseline visual field deficit (N=941)				0.626
No	286 (30.4)	191 (29.9)	95 (31.5)	
Yes	655 (69.6)	448 (70.1)	207 (68.5)	
SBP, mmHg (N=953)	157 (139-174)	157 (140-174)	156 (137-174)	0.230
DBP, mmHg (N=948)	85 (75-95)	86 (76-96)	84 (73-94)	0.046
Vascular risk factors				
Hypertension	816 (77.1)	541 (77.8)	275 (75.6)	0.400
Atrial fibrillation	307 (29.0)	193 (27.8)	114 (31.3)	0.227
Diabetes mellitus	294 (27.8)	207 (29.8)	87 (23.9)	0.042
Hyperlipidemia (N=1,057)	521 (49.3)	368 (53.0)	153 (42.2)	0.001
Current Smoker (N=981)	187 (19.1)	141 (21.0)	46 (14.8)	0.020
Prior Stroke (N=1,045)	191 (18.3)	145 (20.9)	46 (13.1)	0.002
Peripheral Artery Disease (N=958)	57 (6.0)	41 (6.2)	16 (5.4)	0.659
Dialysis (N=979)	38 (3.9)	22 (3.3)	16 (5.3)	0.133
Oral anticoagulation (N=1,036)	152 (14.7)	92 (13.3)	60 (17.3)	0.086
Statin (N=999)	340 (34.0)	233 (34.7)	107 (32.7)	0.541

Table S2A. Baseline characteristics, metrics, and outcomes of patients with P1 and P2 occlusion of posterior cerebral artery (PCA) occlusion treated with Medical Management (MM) vs. Endovascular thrombectomy (EVT) continued.

	Overall (N=1,059)	MM (N=695)	EVT (N=364)	*P*
Imaging and clot location				
pc-ASPECTS	9 (9-10)	9 (9-10)	10 (9-10)	0.001
Baseline imaging modality (N=1,048)				
CT	953 (90.9)	637 (91.7)	316 (89.5)	0.255
MRI	254 (24.2)	205 (29.5)	49 (13.9)	<0.0001
Perfusion-imaging done (CT or MRI)	534 (51.0)	327 (41.1)	207 (58.6)	0.0004
Mismatch ratio > 1.2[+](N=408)	342 (83.8)	189 (77.1)	153 (93.9)	<0.0001
Occlusion site				<0.0001
P1	469 (44.3)	260 (37.4)	209 (57.4)	
P2	590 (55.7)	435 (62.6)	155 (42.6)	
Time metrics and procedural factors				
IV tPA				0.494
No	622 (58.7)	403 (58.0)	219 (60.2)	
Yes	437 (41.3)	292 (42.0)	145 (39.8)	
Time to treatment*, h (N=894)	3.3 (2.0-7.7)	3.0 (1.8-8.5)	3.9 (2.5-7.3)	0.010
Time to treatment*, h (N=894)				0.837
0 - <6	616 (68.9)	390 (68.7)	226 (69.3)	
6 – 24	278 (31.1)	178 (31.3)	100 (30.7)	
Number of passes (N=331)	-	-	1 (1-2)	
Recanalization				
TICI 0-2a	-	-	77 (21.2)	
TICI 2b	-	-	55 (15.1)	
TICI 2c	-	-	34 (9.3)	
TICI 3	-	-	198 (54.4)	
First pass EVT method (N=320)				
Stent retriever	-	-	41 (12.8)	
Contact Aspiration	-	-	105 (32.8)	
Combined technique	-	-	169 (52.8)	
IA lytic	-	-	5 (1.6)	
Other				
Stroke etiology (N=1,054)				0.001
Large artery atherosclerotic	162 (15.4)	82 (11.9)	80 (22.0)	
Cardioembolic	432 (41.0)	297 (43.0)	135 (37.1)	
Small vessel atherosclerotic	24 (2.3)	17 (2.5)	7 (1.9)	
Other determined	85 (8.1)	58 (8.4)	27 (7.4)	
Undetermined	351 (33.3)	236 (34.2)	115 (31.6)	

Continuous variables are reported as median and interquartile range, categorical variables are reported as number of patients and column percentages.

Abbreviations: MM, Medical Management; EVT, Endovascular Therapy; N, the total number of patients; mRS, modified Rankin Scale; NIHSS, National Institutes of Health Stroke Scale; SBP, systolic blood pressure; DBP, diastolic blood pressure; pc-ASPECTS, posterior circulation Acute Stroke Prognosis Early CT Score; CT, computed tomography; MRI, Magnetic resonance imaging;

IV tPA, intravenous tissue-type plasminogen activator; TICI, Treatment in Cerebral Infarction; IA, intra-arterial.

+Calculated as the ratio between hypoperfusion volume and core volume.

*Time from symptom onset or last known well if unwitnessed onset, to administration of IVT or groin puncture for those treated with EVT.

Table S2B. Outcomes of patients with P1 and P2 occlusion of posterior cerebral artery (PCA) occlusion treated with Medical Management (MM) vs. Endovascular thrombectomy (EVT).

	Overall (N=1,059)	MM (N=695)	EVT (N=364)	Univariable comparison P	Univariable model OR (95% CI)	Multivariable model OR (95% CI)	IPTW model OR (95% CI)
mRS, 3 months (N=959)	2 (1-4)	2 (1-3)	2 (1-4)	0.466	0.91 (0.65-1.28)	1.02 (0.71-1.46)	1.09 (0.75-1.59)
mRS 0-1 or RbR, 3 months (N=959)	336 (35.0)	220 (34.8)	116 (35.5)	0.838	1.03 (0.72-1.48)	1.34 (0.90-2.0)	1.29 (0.84-1.97)
mRS 0-2 or RbR, 3 months (N=959)	536 (55.9)	362 (57.3)	174 (53.2)	0.229	0.85 (0.61-1.19)	1.01 (0.67-1.52)	1.04 (0.71-1.54)
NIHSS change (N=1,002)[+]	2 (0-4)	1 (0-3)	3 (0-7)	<0.0001			
NIHSS change ≥2 (N=1,002)	508 (50.7)	298 (44.3)	210 (63.8)	<0.0001	2.22 (1.72-2.86)	1.69 (1.25-2.26)	1.62 (1.23-2.12)
HBC ICH (N=856)	169 (19.7)	98 (17.7)	71 (23.4)	0.045			
Fatal ICH (N=1,001)	11 (1.1)	3 (0.45)	8 (2.4)	0.008			
sICH (N=1,054)	35 (3.3)	11 (1.6)	24 (6.7)	<.0001	4.44 (2.02-9.75)*	4.60 (2.07-10.22)*	5.20 (2.25-12.02)*
Vision recovery (N=233/655)[†]				0.0004			
Complete	107 (45.9)	70 (38.9)	37 (69.8)				
Partial	20 (8.6)	16 (8.9)	4 (7.6)				
Same	89 (38.2)	81 (45.0)	8 (15.1)				
Worse	17 (7.3)	13 (7.2)	4 (7.6)				
Mortality (1,058)	72 (6.8)	31 (4.5)	41 (11.3)	<0.0001	2.73 (1.65-4.52)*	2.94 (1.75-4.94)*	2.14 (1.20-3.80)*

Continuous variables are reported as median and interquartile range. Categorical variables are reported as number of patients and percentages.
Abbreviations: MM, Medical Management; EVT, Endovascular Therapy; N, the total number of patients; OR, odds ratio; CI, confidence interval; IPTW, inverse probability of treatment weighting; mRS, modified Rankin Scale; RbR=Return to baseline Rankin; NIHSS, National Institutes of Health Stroke Scale; HBC, Heidelberg Bleeding Classification; ICH, Intracerebral hemorrhage; sICH, symptomatic intracranial hemorrhage.
[+]Change in NIHSS is defined as NIHSS at admissions – NIHSS at discharge. A positive score means NIHSS at discharge is < NIHSS at admission.
[*] Significant at p<0.05 level
†Of 655 with visual field defects at baseline, information on vision recovery was available for 233. 57 were deceased, while 365 were missing information.
Note: The multivariate model and IPTW analysis account for the following variables: age, sex, baseline NIHSS, baseline mRS, year of treatment, pc-ASPECTS, hypertension, atrial fibrillation, diabetes mellitus, IV tPA, and occlusion site.

Table S3. Multivariable logistic regression, and IPTW evaluation of outcomes after EVT vs. MM: Analysis stratified by baseline NIHSS.

				Multivariable model			IPTW model		
		N	Event (%)*	OR (95% CI), *P*, *P* for interaction					
**Ordinal mRS favorable shift**									
0-6	MM	378	2 (1-3)	referent		0.312	referent		0.792
	EVT	121	2 (1-3)	0.81 (0.47-1.42)	0.470		1.0 (0.51-1.96)	0.998	
>6	MM	254	3 (2-5)	referent			referent		
	EVT	206	3 (1-5)	1.15 (0.78-1.68)	0.486		1.13 (0.70-1.82)	0.615	
**Excellent outcome (mRS 0-1 or RbR at 3 months)**									
0-6	MM	378	175 (46.3)	referent		0.069	referent		0.196
	EVT	121	53 (43.8)	0.90 (0.49-1.64)	0.729		1.06 (0.56-2.04)	0.850	
>6	MM	254	45 (17.7)	referent			referent		
	EVT	206	63 (30.6)	2.01 (1.22-3.31)	0.006		1.80 (1.12-2.89)	0.015	
**Functional independence (mRS 0-2 or RbR at 3 months)**									
0-6	MM	378	281 (74.3)	referent		0.010	referent		0.054
	EVT	121	79 (65.3)	0.55 (0.30-1.0)	0.048		0.66 (0.36-1.21)	0.176	
>6	MM	254	81 (31.9)	referent			referent		
	EVT	206	95 (46.1)	1.64 (1.08-2.51)	0.021		1.45 (0.94-2.24)	0.092	
**Decrease in NIHSS by ≥2 points**									
0-6	MM	409	130 (31.8)	referent		0.399	referent		0.003
	EVT	126	60 (47.6)	1.74 (1.23-2.45)	0.002		1.77 (1.17-2.69)	0.007	
>6	MM	264	168 (63.6)	referent			referent		
	EVT	203	150 (73.9)	1.38 (0.95-2.02)	0.095		1.28 (0.87-1.89)	0.210	
**sICH**									
0-6	MM	418	4 (0.96)	referent		0.467	referent		0.371
	EVT	131	7 (5.3)	6.62 (1.32-33.22)	0.022		6.71 (1.40-32.17)	0.017	
>6	MM	276	7 (2.5)	referent			referent		
	EVT	229	17 (7.4)	3.36 (1.54-7.32)	0.002		3.64 (1.55-8.52)	0.003	
**Mortality**									
0-6	MM	418	4 (0.96)	referent		0.044	referent		0.002
	EVT	133	8 (6.0)	7.95 (3.11-20.28)	<0.0001		7.27 (2.55-20.73)	0.0002	
>6	MM	277	27 (9.8)				referent		
	EVT	230	33 (14.4)	1.98 (1.08-3.65)	0.028		2.0 (1.06-3.80)	0.033	

Abbreviations: IPTW, inverse probability of treatment weighting; N, the total number of patients; OR, odds ratio; CI, confidence interval; mRS, modified Rankin Scale; MM, medical management; EVT, Endovascular therapy; RbR=Return to baseline Rankin; NIHSS, National Institutes of Health Stroke Scale; sICH, symptomatic intracranial hemorrhage.

Note: The multivariate model and IPTW analysis account for the following variables: age, sex, baseline mRS, year of treatment, pc-ASPECTS, hypertension, atrial fibrillation, diabetes mellitus, IV tPA and occlusion site.

*For Ordinal mRS outcome, the median value is displayed.

Table S4. Baseline characteristics, metrics, and outcomes of patients with posterior cerebral artery (PCA) occlusion by occlusion site.

	P1<br>(N=469)	P2<br>(N=590)	*P*
Demographics			
Age, yearr	75 (65-82)	74 (62-81)	0.060
Sex			0.394
Male	262 (55.9)	345 (58.5)	
Female	207 (44.1)	245 (41.5)	
Year of treatment (2000s)	19 (17-20)	19 (17-20)	0.149
Clinical characteristics			
Transfer (N=913)			0.002
Local	340 (79.8)	426 (87.5)	
Transfer	86 (20.2)	61 (12.5)	
Baseline mRS			0.352
0	254 (54.2)	349 (59.2)	
1	98 (20.9)	114 (19.3)	
2	64 (13.7)	75 (12.7)	
3	53 (11.3)	52 (8.8)	
Baseline NIHSS	8 (4-13)	5 (3-8)	<0.0001
Baseline NIHSS			<0.0001
0-6	190 (40.5)	361 (61.2)	
7-19	226 (48.2)	203 (34.4)	
≥20	53 (11.3)	26 (4.4)	
Baseline visual field deficit (N=941)			0.001
No	149 (36.1)	137 (26.0)	
Yes	264 (63.9)	391 (74.1)	
SBP, mmHg (N=953)	156 (138-175)	158 (140-173)	0.584
DBP, mmHg (N=948)	85 (75-96)	86 (76-95)	0.634
Vascular risk factors			
Hypertension	378 (80.6)	438 (74.2)	0.015
Atrial fibrillation	138 (29.4)	169 (28.6)	0.781
Diabetes mellitus	141 (30.1)	153 (25.9)	0.136
Hyperlipidemia (N=1,057)	216 (46.3)	305 (51.7)	0.079
Current Smoker (N=981)	83 (18.6)	104 (19.5)	0.719
Prior Stroke (N=1,045)	98 (20.9)	93 (16.1)	0.045
Peripheral Artery Disease (N=958)	24 (5.7)	33 (6.2)	0.760
Dialysis (N=979)	16 (3.6)	22 (4.1)	0.720
Oral anticoagulation (N=1,036)	66 (14.4)	86 (14.9)	0.812
Statin (N=999)	156 (35.0)	184 (33.3)	0.572

Table S4. Baseline characteristics, metrics, and outcomes of patients with posterior cerebral artery (PCA) occlusion by occlusion site continued.

	P1 (N=469)	P2 (N=560)	*P*
Imaging and clot location			
pc-ASPECTS	9 (8-10)	9 (9-10)	0.004
Baseline imaging modality (N=1,048)			
CT	412 (87.9)	541 (93.4)	0.002
MRI	134 (28.6)	120 (20.7)	0.003
Perfusion-imaging done (CT or MRI)	226 (48.2)	308 (53.2)	0.107
Mismatch ratio > 1.2^{+} (N=408)	141 (82.5)	201 (84.8)	0.524
Time metrics and procedural factors			
IV tPA			0.089
No	289 (61.6)	333 (56.4)	
Yes	180 (38.4)	257 (43.6)	
Treatment			<0.001
MM	234 (55.2)	398 (74.4)	
EVT	190 (44.8)	137 (25.6)	
Time to treatment*, h (N=894)	3.5 (1.9-8.0)	3.3 (2-7.3)	0.573
Time to treatment*, h (N=894)			0.600
0 - <6	261 (68.0)	355 (69.6)	
6 – 24	123 (32.0)	155 (30.4)	
Number of passes (N=331)	1 (1-2)	1 (1-2)	0.361
Recanalization (N=406)			0.475
TICI 0-2a	57 (25.7)	46 (25.0)	
TICI 2b	37 (16.7)	22 (12.0)	
TICI 2c	16 (7.2)	18 (9.8)	
TICI 3	112 (50.5)	98 (53.3)	
First pass EVT method (N=320)			0.314
Stent retriever	23 (12.4)	18 (13.3)	
Contact Aspiration	61 (33.0)	44 (32.6)	
Combined technique	96 (51.9)	73 (54.1)	
IA lytic	5 (2.7)	0 (0.0)	
Other			
Stroke etiology (N=1,054)			0.414
Large artery atherosclerotic	81 (17.4)	81 (13.8)	
Cardioembolic	194 (41.6)	238 (40.5)	
Small vessel atherosclerotic	10 (2.2)	14 (2.4)	
Other determined	33 (7.1)	52 (8.8)	
Undetermined	148 (31.8)	203 (34.5)	

Continuous variables are reported as median and interquartile range. Categorical variables are reported as number of patients and percentages.

Abbreviations: N, the total number of patients; mRS, modified Rankin Scale; NIHSS, National Institutes of Health Stroke Scale; SBP, systolic blood pressure; DBP, diastolic blood pressure; pc-ASPECTS, posterior circulation Acute Stroke Prognosis Early CT Score; CT, computed tomography; MRI, Magnetic resonance imaging; IV tPA, intravenous tissue-type plasminogen activator; MM, Medical Management; EVT, Endovascular Therapy; TICI, Treatment in Cerebral Infarction; IA, intra-arterial.

[+]Calculated as the ratio between hypoperfusion volume and core volume.
*Time from symptoms onset or last known well if unwitnessed onset, to administration of IVT or groin puncture for those treated with EVT.

Table S5. Multivariable logistic regression, and IPTW evaluation of outcomes after EVT vs. MM: Analysis stratified by P1 and P2 occlusion of posterior cerebral artery.

				Multivariable model			IPTW model		
		N	Event (%)*	OR (95% CI), *P*, *P* for interaction					
**Ordinal mRS favorable shift**									
P1	MM	234	3 (2-4)	referent		0.188	referent		0.129
	EVT	190	3 (1-5)	0.84 (0.53-1.32)	0.438		0.82 (0.57-1.19)	0.296	
P2	MM	398	2 (1-3)	referent			referent		
	EVT	137	2 (1-4)	1.24 (0.81-1.89)	0.324		1.33 (0.78-2.29)	0.294	
**Excellent outcome (mRS 0-1 or RbR at 3 months)**									
P1	MM	234	68 (29.1)	referent		0.987	referent		0.721
	EVT	190	61 (32.1)	1.34 (0.82-2.18)	0.245		1.36 (0.90-2.05)	0.147	
P2	MM	398	152 (38.2)	referent			referent		
	EVT	137	55 (40.2)	1.34 (0.84-2.15)	0.217		1.24 (0.71-2.17)	0.441	
**Functional independence (mRS 0-2 or RbR at 3 months)**									
P1	MM	234	113 (48.3)	referent		0.594	referent		0.531
	EVT	190	89 (46.8)	0.93 (0.57-1.52)	0.759		0.95 (0.66-1.35)	0.762	
P2	MM	398	249 (62.6)	referent			referent		
	EVT	137	85 (62.0)	1.11 (0.64-1.92)	0.723		1.13 (0.63-2.04)	0.686	
**Decrease in NIHSS by ≥2 points**									
P1	MM	245	118 (48.2)	referent		0.478	referent		0.350
	EVT	184	117 (63.6)	1.50 (0.95-2.35)	0.082		1.45 (0.92-2.29)	0.108	
P2	MM	428	180 (42.1)	referent			referent		
	EVT	145	93 (64.1)	1.89 (1.26-2.84)	0.002		1.75 (1.17-2.63)	0.007	
**sICH**									
P1	MM	260	3 (1.2)	referent		0.276	referent		0.786
	EVT	208	15 (7.2)	7.57 (2.11-27.18)	0.002		6.90 (1.97-24.24)	0.003	
P2	MM	434	8 (1.8)	referent			referent		
	EVT	152	9 (5.9)	3.25 (1.06-9.99)	0.040		4.27 (1.53-11.91)	0.006	
**Mortality**									
P1	MM	260	15 (5.8)	referent		0.507	referent		0.145
	EVT	209	28 (13.4)	3.49 (1.55-7.89)	0.003		2.39 (1.06-5.37)	0.035	
P2	MM	435	16 (3.7)				referent		
	EVT	154	13 (8.4)	2.35 (1.19-4.63)	0.014		1.88 (0.87-4.05)	0.108	

Abbreviations: IPTW, inverse probability of treatment weighting; N, the total number of patients; OR, odds ratio; CI, confidence interval; mRS, modified Rankin Scale; MM, medical management; EVT, Endovascular therapy; RbR=Return to baseline Rankin; NIHSS, National Institutes of Health Stroke Scale; sICH, symptomatic intracranial hemorrhage.
Note: The multivariate model and IPTW analysis account for the following variables: age, sex, baseline NIHSS, baseline mRS, year of treatment, pc-ASPECTS, hypertension, atrial fibrillation, diabetes mellitus, and IV tPA.
*For Ordinal mRS outcome, the median value is displayed.

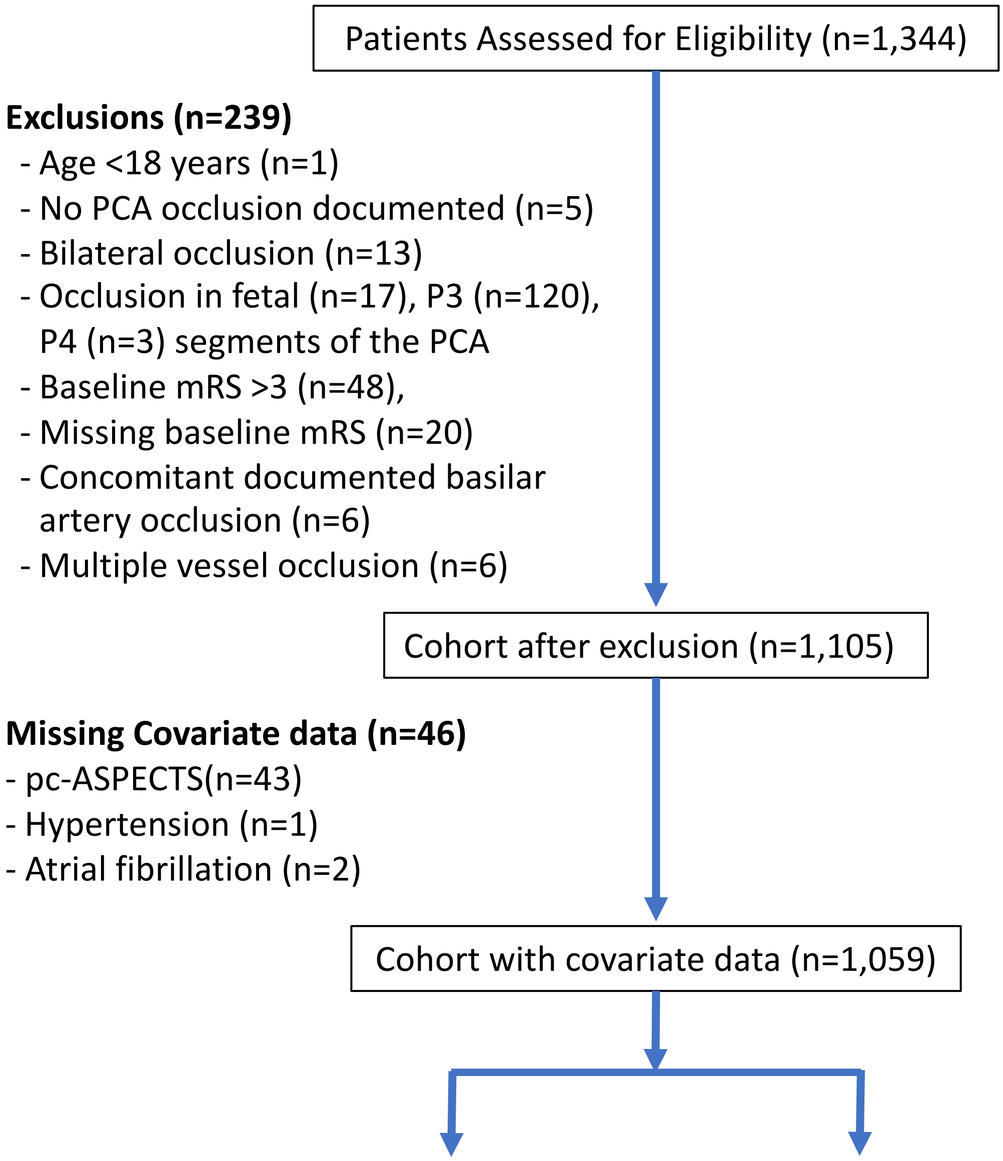

Outcome	Available	Medical Management (n=695)	Endovascular therapy (n=364)	Missing
3-month mRS	959	632	327	100 (9%)
NIHSS change	1002	673	329	57 (5%)
SICH	1054	694	360	5 (0.5%)
Mortality	1058	695	364	1 (0.1%)

Figure S2. *Interaction between treatment and NIHSS as continuous variables (inverse probability of treatment weighting models)*

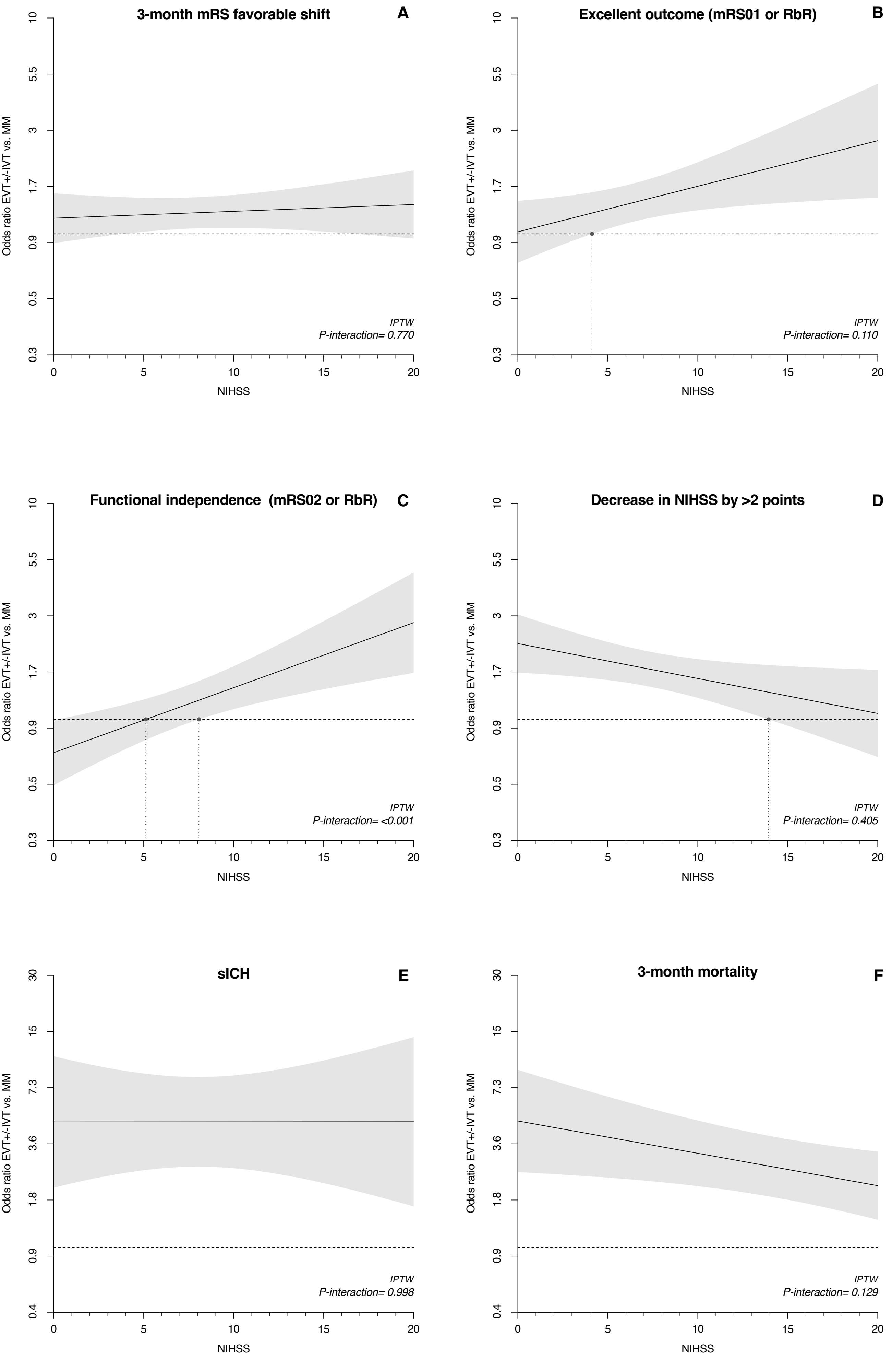

Supplement: Supplementary file 1 [file str-55-1787-s001.pdf]
